# CIMIDx: Prototype for a Cloud-Based System to Support Intelligent Medical Image Diagnosis With Efficiency

**DOI:** 10.2196/medinform.3709

**Published:** 2015-03-27

**Authors:** Selvaraj Rani Bhavani, Jagatheesan Senthilkumar, Arul Gnanaprakasam Chilambuchelvan, Dhanabalachandran Manjula, Ramasamy Krishnamoorthy, Arputharaj Kannan

**Affiliations:** ^1^Department of Computer Science and EngineeringCollege of EngineeringAnna UniversityChennai, TamilnaduIndia; ^2^Department of Computer Science and EngineeringBharathidasan Institute of Technology, BIT CampusAnna UniversityTiruchirapalli, TamilnaduIndia; ^3^Department of Information Science and TechnologyCollege of EngineeringAnna UniversityChennai, TamilnaduIndia

**Keywords:** association rules, cloud computing, breast cancer, pre-processing, segmentation, feature extraction, intelligent system, UDDI, SOAP, Web-based intervention, medical diagnosis

## Abstract

**Background:**

The Internet has greatly enhanced health care, helping patients stay up-to-date on medical issues and general knowledge. Many cancer patients use the Internet for cancer diagnosis and related information. Recently, cloud computing has emerged as a new way of delivering health services but currently, there is no generic and fully automated cloud-based self-management intervention for breast cancer patients, as practical guidelines are lacking.

**Objective:**

We investigated the prevalence and predictors of cloud use for medical diagnosis among women with breast cancer to gain insight into meaningful usage parameters to evaluate the use of generic, fully automated cloud-based self-intervention, by assessing how breast cancer survivors use a generic self-management model. The goal of this study was implemented and evaluated with a new prototype called “CIMIDx”, based on representative association rules that support the diagnosis of medical images (mammograms).

**Methods:**

The proposed Cloud-Based System Support Intelligent Medical Image Diagnosis (CIMIDx) prototype includes two modules. The first is the design and development of the CIMIDx training and test cloud services. Deployed in the cloud, the prototype can be used for diagnosis and screening mammography by assessing the cancers detected, tumor sizes, histology, and stage of classification accuracy. To analyze the prototype’s classification accuracy, we conducted an experiment with data provided by clients. Second, by monitoring cloud server requests, the CIMIDx usage statistics were recorded for the cloud-based self-intervention groups. We conducted an evaluation of the CIMIDx cloud service usage, in which browsing functionalities were evaluated from the end-user’s perspective.

**Results:**

We performed several experiments to validate the CIMIDx prototype for breast health issues. The first set of experiments evaluated the diagnostic performance of the CIMIDx framework. We collected medical information from 150 breast cancer survivors from hospitals and health centers. The CIMIDx prototype achieved high sensitivity of up to 99.29%, and accuracy of up to 98%. The second set of experiments evaluated CIMIDx use for breast health issues, using *t* tests and Pearson chi-square tests to assess differences, and binary logistic regression to estimate the odds ratio (OR) for the predictors’ use of CIMIDx. For the prototype usage statistics for the same 150 breast cancer survivors, we interviewed 114 (76.0%), through self-report questionnaires from CIMIDx blogs. The frequency of log-ins/person ranged from 0 to 30, total duration/person from 0 to 1500 minutes (25 hours). The 114 participants continued logging in to all phases, resulting in an intervention adherence rate of 44.3% (95% CI 33.2-55.9). The overall performance of the prototype for the good category, reported usefulness of the prototype (*P*=.77), overall satisfaction of the prototype (*P*=.31), ease of navigation (*P*=.89), user friendliness evaluation (*P*=.31), and overall satisfaction (*P*=.31). Positive evaluations given by 100 participants via a Web-based questionnaire supported our hypothesis.

**Conclusions:**

The present study shows that women felt favorably about the use of a generic fully automated cloud-based self- management prototype. The study also demonstrated that the CIMIDx prototype resulted in the detection of more cancers in screening and diagnosing patients, with an increased accuracy rate.

## Introduction

The past few decades have seen major advancements in medical science and technology, which have transformed the medical field, and the implications are apparent [1]. However, millions of people across the world do not have the opportunity to access optimum Web-based medical health care services, and are limited by their cost and accessibility [2]. Information communication technology (ICT) has revolutionized an operating model that presents an opportunity for universal access to medical information at very low cost [3]. However, the existing models or systems face many barriers such as capacity building, accuracy, integration of prevailing health systems, promotion of inter-operability using universal standards, cost, eHealth observatory, and security, and do not provide free health services [4]. To overcome these barriers, cloud computing is emerging as a new way of delivering computing resources and health services.

Medical experts believe that cloud computing can improve health care services, benefit health care research, and change the face of health information technology [5]. Cloud computing refers to an on-demand, self-service in the cloud Internet infrastructure, that enables the user to access computing resources anytime, from anywhere in the world, with the help of the Internet [6]. The cloud is a new model of delivering computing resources to health care service provider industries, for the development of medical applications, which includes Microsoft HealthVault and Google Health platform [7]. Compared with conventional computing, the cloud model provides three advantages: (1) massive computing resources available on demand, (2) elimination of an upfront commitment by users, and (3) payment for use on a short-term basis as needed [8]. Health care, as with any other service operation, requires continuous and systematic innovation in order to remain cost effective, efficient, timely, and provide high-quality services [5]. The biomedical informatics community, especially consortiums that share data and applications, can take advantage of the new computing paradigm [9]. Anderson et al [10] indicated that data-handling problems, complexity, and expensive or unavailable computational solutions to research problems are major issues in biomedical research data management and analysis.

Some of the commercially available cloud platforms include Amazon Elastic Compute Cloud (EC2) [11], Google App Engine [12], and Microsoft Windows Azure [13]. The cloud model is composed of three main services depending on the capability and availability to support Web-based health services, such as (1) Infrastructure as a Service, (2) Platform as a Service, and (3) Software as a Service. In addition, cloud computing has special features for clients (radiologists, physicians, researchers, and patients), aiming to reduce the burden of heavy investments and to utilize resource outsourcing, software, hardware, automated resource management, parallel computing, virtualization, and utility computing [9].

The medical image datasets are usually large scale and distributed in different hospitals and, at the same time, the physicians who are skilled in special diseases are spread across the globe [14-16]. In the past, the medical experts collected the images that the hospital provided, analyzed the images, and obtained results, which was time-consuming. They could not get better analytic results as the asynchronous collaboration could not give the physicians real-time feedback [15]. Synchronous collaboration among physicians should be a more effective way to share knowledge and experience during the process of analysis. It is a challenge to provide a medical image collaborative analysis system (MICAS), which can enable physicians to do synchronous collaborative analysis on medical images over geographic distances [16]. Web services are platform independent and provide the facility for experts and patients to access medical services in the cloud environment [14,16]. Medical Web services are important applications running on the cloud server and these services can be accessed by clients through computers from remote places anywhere in the world [14-16]. A medical Web service can provide guidance to clients and offers services through the Internet in real time. The client can access the Web service with minimum software or even none, since many of these applications are accessible using a Web browser.

Breast cancer is by far the most common cancer diagnosed in women worldwide [17-20]. An estimated 1.38 million women across the world were diagnosed with breast cancer in 2008, accounting for nearly a quarter (23%) of all cancers diagnosed in women (11% of the total in men and women). The incidence is generally high in developed countries and markedly lower in developing countries, though the difference in population sizes means that an approximately equal number of cases were diagnosed in the developed and developing regions in 2008 (around 690,00 cases each). Breast cancer incidence has increased in most countries worldwide in recent decades, with the most rapid increase occurring in many of the developing countries [18,20]. Breast cancer is the second-most common and leading cause of cancer death in women in the world. Women with breast cancer in their family are more susceptible to developing breast cancer. The risk also increases by age. It has become a major health issue in the world over the past 50 years and its incidence has increased in recent years [17]. Early detection is an effective way to diagnose and manage breast cancer. Mammograms can help early detection or diagnosis of breast cancer and increase patient survival rates. A vast number of medical images are generated daily in hospitals and medical centers. Consequently, radiologists have to analyze more and more images manually. After analyzing a small number of images, the process of diagnosing becomes more complicated and leads to susceptible errors. The computerized analysis of medical images has evolved from automated computer-aided detection (CADe) or computer-aided diagnosis (CADx) systems, where radiologists use computer output as a “second opinion” to assist them, speeding up the diagnosing task and bringing more confidence to it [21-26]. CADe or CADx systems have been successfully introduced in many hospitals and specialized clinics, to provide quick access to screening. The CADe or CADx system can play an important role in the early detection or diagnosis of breast cancer, and reduce the death rate among women due to breast cancer. The CADe system in detection work-up usually involves having the computer extract the margin of the lesion from the surrounding parenchyma, extract characteristics (features) of the lesions, merge these computer-extracted features into an estimate of the probability of the mammogram abnormalities [21]. The primary goal of CADe is to increase detection of the disease by reducing the false negative rate, which could be due to observational oversight [22]. The CADx systems in diagnostic work-up involve the computer identifying the region of interest (ROI) in the lesion, extract the visual contents (features) of the lesions, and merge these computer-extracted features with diagnostic keywords. The merged features are given to the feature selection and discretization method to identify the consistent features and further estimate the probability of mammogram abnormalities [23-26]. In the medical domain, the objective of a CADx system is to aid the specialist in the medical diagnosis process [23-26], retrieving relevant [26] past cases with images revealing proven pathology, along with the corresponding associated clinical diagnoses and other information. Recently, the CADx system has begun supporting experts and patients in analyzing digital images to identify possible diseases, via the cloud environment [27]. Thus, building a CADx system in health care is becoming highly important and a priority for research in hospitals and medical centers [21-27].

**Figure 1:**
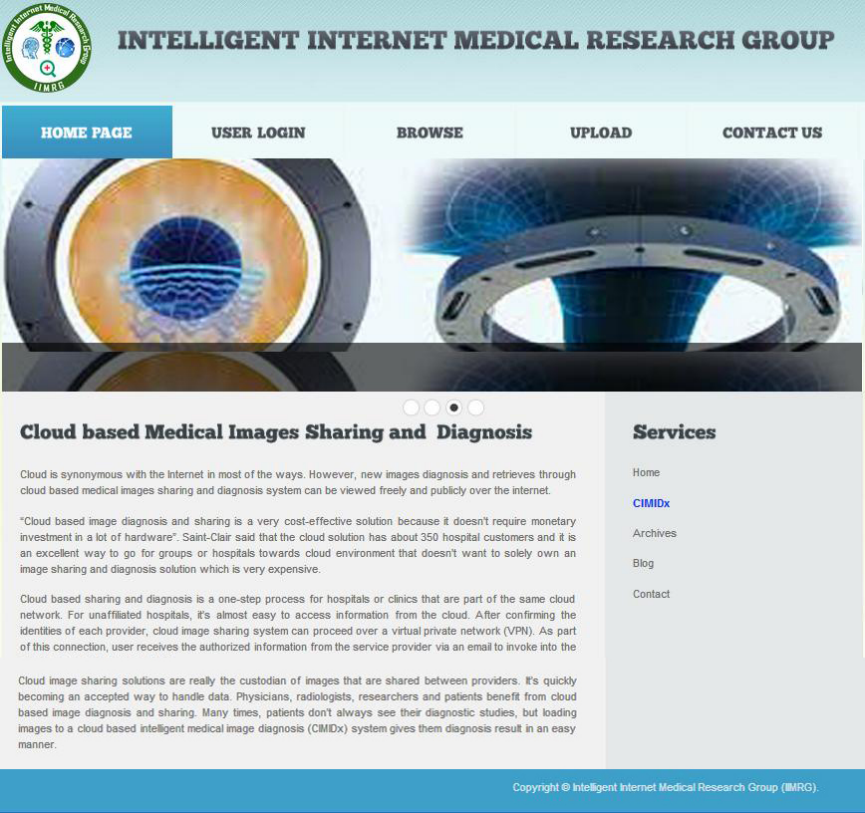
Intelligent Internet Medical Research Group.

CADx is a complex procedure that requires several processing phases in breast cancer images, such as pre-processing, segmentation [28-31], feature extraction [32-36], feature dimensional reduction [37-40], association rule mining [41-44], and classification [25,26]. To provide these functionalities in a separate way in the cloud environment is very difficult. Web services that enable users to access heterogeneous, distributed resources provide easier integration and interoperability between data and applications in the cloud environment [45]. The cloud provides the functionality to access computational resources for image processing [46], image retrieval [48], and mining biomedical data [48]. Web services are accessed through the HTTP/HTTPS protocols, and utilize Extensible Mark-up Language (XML) for data exchange [49]. The interaction among the Web service components exploits the Universal Description, Discovery and Integration (UDDI) registry. The service provider defines a reference to its Web service, using the Web service description language (WSDL). The WSDL document is published in the UDDI registry; the service consumer can search the registry and retrieve the WSDL reference to the Web services. The service consumer, using the information stored inside the WSDL document, contacts the Web service and issues a service request in the cloud environment. Figure 1 shows the cloud-based medical images sharing and diagnosis framework.

In this research, new intelligent medical image diagnostic Web services were developed and deployed in the cloud, called “CIMIDx” (Cloud-Based System Support Intelligent Medical Image Diagnosis). The proposed CIMIDx prototype provides the facility for clients to support the diagnosis of a medical image in the heterogeneous environment. It consists of two main service models, namely training and test service models. These service models are deployed in the UDDI cloud server, by which patients, radiologists, physicians, and researchers (both engineering and medical) make use of the CIMIDx prototype. The proposed framework facilitates cross-platform application and makes access to the CIMIDx prototype easy for the diagnosis of the medical image. This proposed approach was applied to 150 client images, and the result shows high sensitivity (up to 99.29%) and accuracy (up to 98%).

## Methods

### Research Design

The proposed study was conducted in May 2013 in three phases. In the first phase, researchers focused on general breast cancer image diagnosis, treatment experiences, and the reactions of the medical image diagnosis participants. The second phase of the study was a usability test of the site that focused on participant reactions about the CIMIDx prototype. The third phase of this study was based on the user reactions about the general idea for the CIMIDx prototype, and further refinement with the new technologies. Based on user reactions, the introductory site content and approach were revised. In this research, we conducted two different sets of experiments for the proposed CIMIDx prototype. The first set of experiments aimed at evaluating the classification performance of the client’s use of the CIMIDx framework. The second set of experiments aimed at validating the usability of the CIMIDx for breast health issues. We used *t* tests and Pearson chi-square tests to assess the differences and binary logistic regression to estimate the odds ratio (OR) for the predictors of CIMIDx for breast health issues.

### CIMIDx Architecture

**Figure 2:**
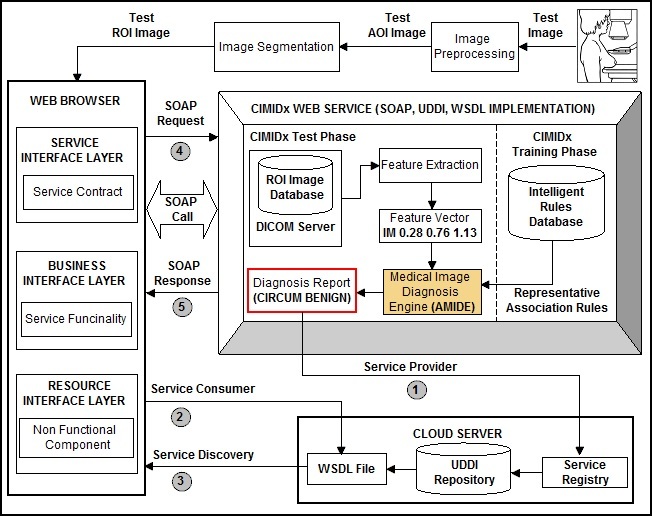
The proposed CIMIDx architecture.

The proposed CIMIDx framework combines visual features, automatically extracted from the medical image, with the high-level knowledge given by the specialist about the training images, to search for patterns. The CIMIDx prototype consists of two main service models, namely, training and test models. Each training image is associated with a set of keywords or classes (keywords are representative words given by a specialist to use in the diagnosis of a medical image). Figure 2 shows the proposed CIMIDx architecture.

### CIMIDx Web Service Model

#### Overview

The CIMIDx Web service framework is cloud-based, so that the client side contains diagnostic Web pages shown to the user to test the new medical image, without intervention anywhere in the world. The developer has the ability to train and test the medical image in the cloud environment. The client has the ability to test a new image on his/her computer and receive the diagnosis result from the CIMIDx prototype running on the cloud server. In this research, an integrated approach based CIMIDx training and test Web services framework, which supports the distributed medical image diagnosis, is presented. In the training service model, the CIMIDx prototype has derived new knowledge as representative association rules (intelligent rules) by invoking the following five Web service models, namely, image pre-processing, segmentation, visual feature extraction, feature selection and discretization, and representative association rule mining. In the test service model, the clients are invoked by the CIMIDx test Web services with a new diagnosis image, by using the following four test Web service models viz, image pre-processing, image segmentation, visual feature extraction, and medical image diagnosis engine.

The proposed method facilitates the sharing of resources and infrastructure in the cloud environment. These resources can be located at different nodes that may be accessed through Web services published in the cloud platform. The philosophy of the platform is to minimize the time of the CIMIDx application development, and provide this facility to the client without any intervention. The communication established between the CIMIDx service providers’ application and the cloud server (where the CIMIDx prototype deployment is accomplished) is done using the Simple Object Access Protocol (SOAP) protocol. Hence, the implemented CIMIDx training and test Web service applications are able to publish using Web services. The CIMIDx Web service framework includes four different methods as follows.

1. Central Controller: The Central controller (CC) is responsible for the execution of the CIMIDx method in the task of medical image diagnosis in a cloud environment. It is also responsible for publishing the CIMIDx training and test Web service model, and making the services available to the developer and the clients.

2. Web Browsers: The Web browser is responsible for invoking the CIMIDx prototype through the SOAP call from any computer connected to the Internet. It contains three layers: the service interface layer, business interface layer, and resource interface layer. The components of the service interface layer include the service contract and the service adapter. The service contract defines what operations the CIMIDx service can perform but it does not include any behavior, such as how the operation is actually implemented. This requires defining interfaces, which are groups of operations defined in terms of message exchange patterns. For each operation, the developer defines the message type used in the exchange, and defines each message type in terms of the composable data type. The service adapter implements the service contract that is exposed on an endpoint (commonly referred to as the service host) and is responsible for adapting the endpoint to the underlying business layer.

The business layer consists of three components: business entities, business logic, and business workflows. Business entities are classes that model real-world, domain-specific objects. They are different from the data types used in the service contract because they include behavior and perhaps state. The business layer encapsulates the business entities so that they are not exposed across a service boundary. This ensures more flexibility within each layer. It also gives the opportunity to format the data in different ways, catering to specific integration scenarios. However, this also means that entity translation is necessary for moving between the layers.

The business logic implements the actual business behavior. These classes operate on the business entities to perform a desired action. Some business entities are quite simple while others take advantage of more complex logic.

Business workflows handle long-running processes that require sophisticated message correlation and state management. They are typically implemented with a business process management product. The business layer operates on the underlying resource access layer.

The resource interface layer provides access to both data access logic and service agents. The data access logic provides the opportunity for interacting with the underlying data store while the service agents provide the facility for the developer and client to interact with external Web services. 

3. Web services: A set of Web services (training and test) implements the CIMIDx framework, by invoking the developer and client for an effective diagnosis of the medical image in the cloud environment.

4. CIMIDx Web services: CIMIDx Web services are deployed in the cloud. The developer and the client are able to access the CIMIDx prototype remotely, for diagnosis of the medical image provided by the shared databases within or outside the cloud network.

The CIMIDx framework contains a Web application and uses the interface, which allows accessing the proposed system from any computer connected to the Internet and a Web browser. CIMIDx also includes an application server, which is in charge of the processing methods and the communications with the remote applications that offer them. Communications between user interface and remote CIMIDx services are accomplished through Web languages. The communication between the client and remote CIMIDx services is performed using SOAP messages. Together with Web capabilities, the CIMIDx Web service architecture offers the possibility of integrating algorithms developed in different computer languages, enabling the integration and linkage of already developed libraries. The detailed workflows for the processing of the medical image diagnostic methods, integrated using the CIMIDx Web service, are described below.

#### Service Discovery

The Service Discovery (SD) creates the new service directory in the service agent that acts as a proxy between the service consumer and the service provider. The service agent provides the list of services to the CIMIDx developers and clients, which are retrieved from the repository of the regulating authority. The administrator and the client select the set of services that are needed to refine and diagnose a new image. Based on the selected service, the service agent will send the requested service and its respective method name to the service scheduler. The service discovery acts as an intermediary between the service providers and service requesters. The following steps are involved to create a service directory in the cloud server: (1) service discovery accepts requests from service providers to publish and advertise Web service descriptions, ie, the WSDL file format, and (2) it allows the new service requesters to search the collection of service descriptions contained within the service registry.

The main role of the service registry in the service directory is the matchmaking between service providers and service requesters. Once the match has been found, the interactive processes are carried out directly between the service requester and the service provider.

#### Service Provider

The Service Provider (SP) defines the reference in its Web service, using the WSDL. Once the WSDL document has been published in the UDDI registry, the service consumer can search the registry, and then retrieve the WSDL reference from the Web service in the cloud. The following steps are necessary for creating the training and test service description in the cloud: (1) the service provider develops a new training and test service description for the proposed CIMIDx prototype, such as image pre-processing, segmentation, feature extraction, feature selection and discretization, representative association rule mining, and medical image diagnosis engine, (2) once the CIMIDx training and test services are created and deployed in the cloud (runtime) environment, the developer and client can access the respected services through the Internet, (3) SP publishes the training and test services description into one or more service registries in the cloud, and (4) SP receives the invoking service messages from the service requesters.

#### Service Consumer

The Service Consumer (SC) is a client that invokes the test Web services for the diagnosis of the medical image through the Web browser. Using WSDL, the client issues an XML SOAP request to the developed CIMIDx Web service method and the diagnosis result is obtained as an XML SOAP response. The following steps are involved to develop the service consumer details in the cloud environment: (1) the service consumer (client) of the CIMIDx prototype searches for the WSDL file from the UDDI registry, (2) once the SC finds the WSDL file in the UDDI, it issues an XML SOAP request to the created CIMIDx test Web services method, (3) then the SC invokes the CIMIDx test services sequentially, and performs the medical image diagnosis automatically in the cloud environment, and (4) finally, the XML SOAP response as diagnosis result (keyword) is given to the service consumer.

### CIMIDx Training and Test Web Services

#### Overview

**Figure 3:**
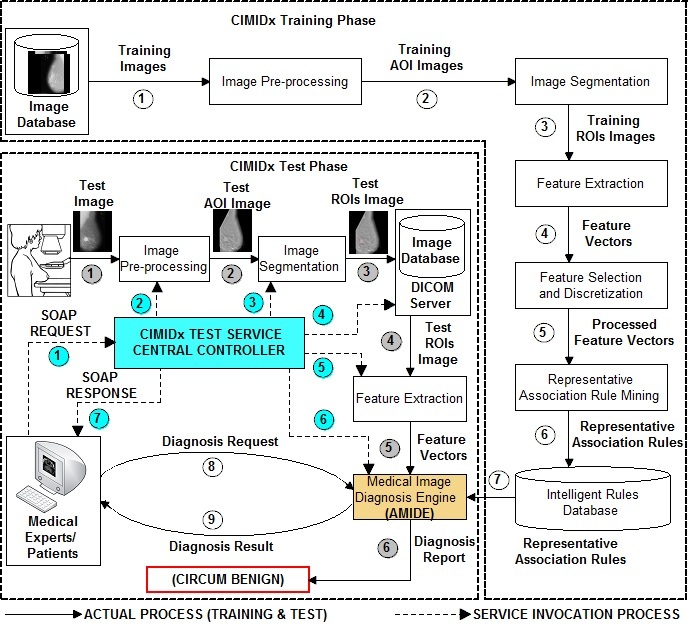
Pipeline of the proposed CIMIDx prototype.

Once the service provider and client receive the WSDL file from the UDDI, the central controller issues the XML SOAP request to assess the CIMIDx prototype, and the XML SOAP response as the diagnosis result. The central controller passes the control between the CIMIDx training and test service model and the client, as shown in Figure 3.

#### CIMIDx Training Web Services

The central controller invokes the CIMIDx training Web services method, which extracts new knowledge as representative association rules to support the diagnosis of the medical image in the cloud environment. The CIMIDx training Web services invoking procedure is as follows.

First, the CC passes the training images to the CIMIDx training Web service, by invoking the image pre-processing Web service model. The fundamental step of an intelligent medical image analysis is image pre-processing, which identifies the area of interest (AOI) in the medical images. The presence of the pectoral muscle in mammograms biases the detection procedure and recommends removing the pectoral muscle during mammogram pre-processing. The proposed image pre-processing method contains two phases, namely breast contour identification and pectoral muscle removal. In the first phase, the proposed method identifies the breast contour (breast profile orientation) with region-based active contour model, with the level set formulation method [31]. The region-based level set method deals with intensity inhomogeneity for the identification of the breast profile orientation in the mammogram image. In the pectoral muscle removal phase, we define an accurate AOI containing the pectoral muscle, after obtaining the breast border in the breast contour identification phase. The proposed method initially defines four control points *x*_1_, *x*_2_, *y*_1_, and *y*_2_, which are used to describe the pectoral muscle region. The control point *x*_1_ is the top-left corner pixel of the breast contour, *x*_2_ is the top-right corner pixel of the breast contour, *y*_1_ lowest pixel on the left edge of the boundary, and *y*_2_ lowest pixel on the right edge of the boundary. Next, the proposed method segments the pectoral muscle region with the Seeded Region Growing (SRG) technique [50]. The proposed method defines two new control points *x*_3_ and *y*_3_ in addition to the four control points *x*_1_, *x*_2_, *y*_1_, and *y*_2_.The control point *x*_3_ is the immediate variations of intensity between *x*_1_ and *x*_2_. The control point *y*_3_ is the immediate variation of intensity between *x*_1_ and *y*_1_. Then the proposed method defines the straight line between the control points *x*_3_ and *y*_3_, using the straight line equation *y*=*mx*+*c*. This method removes the rough edges present in the three control points *x*_1_, *x*_3_, and *y*_3_ in the pectoral muscle boundary. Finally, the proposed image pre-processing method identifies the accurate AOI in the mammogram image. This procedure makes use of these techniques [32-36,56].

Second, the CC passes the training images to the CIMIDx training Web service by invoking the image segmentation Web service model. The image segmentation is achieved in two phases, namely, edge detection and edge refinement. In the edge detection phase, we identified the accurate region edges based on the orthogonal polynomials [28]. The edge detection method performs two different tasks in a single step, such as orthogonal feature components extraction and edge detection. In the orthogonal feature components extraction stage, a class of orthogonal polynomials obtained from the point-spread operators for different sizes of the image window is proposed. A simple computational procedure for constructing a complete set of difference operators from these point-spread operators is employed in the edge detection method. Based on the polynomials’ operator, the edge detection method extracted a set of orthogonal feature components with DC (direct current) energy feature coefficients, AC (alternating component) edge, and texture feature coefficients from the medical image. Then, the extracted orthogonal feature components are utilized to identify the region edges in the medical image. In the edge detection stage, we conducted the Nair test [51] and the *F* test [52] to separate the responses to the edge and noise in the orthogonal feature components, due to the polynomials’ operator. Finally, the image edges are detected by maximizing the signal-to-noise ratio (SNR). The extracted edges are submitted to the edge refinement phase for further identifying the accurate ROI in the image. This procedure makes use of these techniques [28,51-55].

In the edge refinement phase, the edge-based active contour model is devised with a level set formulation method, based on the orthogonal polynomials [28] and level set method (LSM) [54]. The extracted region edges in the edge detection phase are further refined, using a variational level set formulation method [55]. The edge refinement method is a variational level set formulation, in which the regularity of the level set function (LSF) is intrinsically maintained during the level set evolution. The level set evolution is derived as the gradient flow that minimizes the energy functional with a distance regularization term, and an external energy (edge information) that drives the motion of the zero level set toward desired locations. The distance regularization term is defined with a potential function, such that the derived level set evolution has a unique forward-and-backward (FAB) diffusion effect, which is able to maintain the desired shape of the LSF, particularly a signed distance profile near the zero level set. This method yields a new type of level set evolution called edge-based active contour model with level set formulation. The distance regularization effect eliminates the need for reinitialization, and thereby avoids its induced numerical errors. The edge refinement method also allows the use of more general and efficient initialization of the LSF. In its numerical implementation, relatively large time steps can be used in the finite difference scheme to reduce the number of iterations, while ensuring sufficient numerical accuracy. Based on this procedure, the edge-based active contour model with the level set formulation method identifies the accurate ROI in the image. This procedure makes use of these techniques [54,55]. The image segmentation Web services model responds as the ROI image, to the central controller for further visual feature extraction.

Third, the CC passes the ROI image to the visual feature extraction service model. The CIMIDx method extracted 1037-dimensional visual sub-band statistical and spectral orthogonal polynomials based texture features from each image. It includes features generated by the orthogonal polynomials based texture feature (113 features), sub-band statistical and spectral orthogonal polynomials based texture feature (448 features), bivariate discrete orthogonal polynomials based texture feature (336 features), and the gradient gray level co-occurrence probabilities based texture feature (140 features). The visual feature extraction Web service model responses the 1037 texture features to the central controller for further feature selection and discretization. This procedure makes use of these techniques [32-37,56].

Fourth, the CC passes the texture feature vectors to the feature selection and discretization service model. The CIMIDx method uses the NANO algorithm [57] and produces consistent features in the feature database. The NANO algorithm combines feature selection and discretization in a single step, and reduces the mining complexity. This algorithm is employed to solve two problems: feature discretization and selection in a single step. An important contribution is the reduction of irrelevant items to be mined, and the same is achieved with the proposed NANO algorithm. The algorithm NANO selects the relevant features based on the average global inconsistency and average global cut point measures, speeding up the CIMIDx framework. The feature selection and discretization Web service model responses as 135 consistent features to the central controller, for further mining the representative association rules. This procedure makes use of these techniques [57].

Finally, the central controller passes the consistent features to the representative association rule mining service model. The CIMIDx method mines the representative association rules, based on the bounded portion of the density frequency pattern tree and density frequency pattern growth methods. The proposed mining method produces representative association rules to support the diagnosis of the medical image. This model produces representative association rules to the central controller. This procedure makes use of these techniques [58,59].

All of the above processes are executed in a sequential manner. The CC passes the representative association rules as a SOAP response to the developer**.** The extracted representative association rules are then hosted in the cloud server to support the intelligent medical image diagnosis in an efficient manner.

#### CIMIDx Test Web Services

In the test model, the administrator and clients invoked the CIMIDx test Web services with the new image. The CIMIDx test Web services method diagnoses the new image (without the biopsy details) by invoking the following four Web services.

First, the CC passes the test image to the CIMIDx test Web service, by invoking the image pre-processing Web service model for the identification of AOI. The proposed method contains two phases, namely, breast contour identification, and pectoral muscle removal. In the first phase, the proposed method identifies the breast contour (breast profile orientation) based on the LSM [31]. Second, the method segments the pectoral muscle with the breast profile orientation image, using the seeded region growing technique [50]. Finally, the proposed pre-processing method identifies the accurate AOI in the mammogram image. This procedure makes use of these techniques [31,50].

Second, the CC passes a test image to the image segmentation Web service model. The proposed image segmentation method identifies the accurate ROI, based on the orthogonal polynomials and LSM. The proposed image segmentation Web services model responds and passes the ROIs image to the central controller. This procedure makes use of these techniques [28,51-55].

Third, the segmentation method passes the region of interest image to the visual feature extraction method. The ROI image is used for the extraction of visual features automatically. A total of 1037-dimensional visual sub-band statistical and spectral orthogonal polynomials based texture features were computed for each image. It includes features generated by the orthogonal polynomials based texture feature (113 features), sub-band statistical and spectral orthogonal polynomials based texture feature (448 features), bivariate discrete orthogonal polynomials based texture feature (280 features), and the gradient gray level co-occurrence probabilities based texture feature (140 features). This procedure makes use of these techniques detailed in [32-36,56]. These texture features are given to the feature selection and discretization method. The detail of the visual feature extraction process is discussed in visual feature extraction service model.

Finally, the central controller passes the representative association rules with the feature vectors from the test image to the medical image diagnosis Web service model. The CIMIDx model uses the Associative Medical Image Diagnosis Engine (AMIDE) algorithm to classify a new image. The diagnosis result (keyword) is passed to the central controller. All the above processes are executed in a sequential manner. The central controller passes the diagnosis keyword as a SOAP response to the developer and the client**.**

### Associative Medical Image Diagnosis Engine (AMIDE) Algorithm

#### Overview

In this research, a new medical image diagnosis algorithm, called “AMIDE” is presented. In the AMIDE algorithm, it is necessary to clarify some terms. We say that an image matches the set of representative association rules, if the image features satisfy the whole body of the representative association rules (decision rules). An image partially matches a rule, if the image features only satisfy part of the decision rules body. An image does not match a rule, if the image features do not satisfy any part of the decision rules body. AMIDE is a new special classifier that can return multiple keywords (classes) when processing a test image.

#### Condition 1

**Figure 4:**
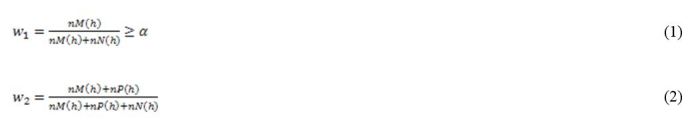
Equations (1) and (2).

**Figure 5:**
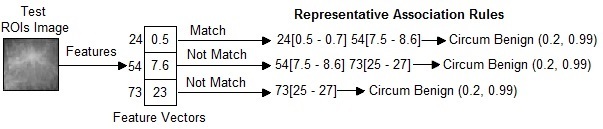
Example to show the calculation of Associative Medical Image Diagnosis Engine (AMIDE) in Condition 1.

The AMIDE algorithm stores all itemsets (set of keywords) *h* belonging to the head of the decision rules in a data structure. An itemset *h* is returned by AMIDE in the suggested diagnosis if the condition stated in (1) Figure 4 is satisfied, where *nM*(*h*) is the number of matches of the itemset *h*, and *nN*(*h*) is the number of non matches. The weight of the itemset is *w*_1_ indicates the strength that an itemset belongs to the diagnosis. The higher the value of the weight, the higher is the confidence that *h* belongs to the diagnosis of the image. A threshold *α* of minimum weight 0<α≤1 is employed to limit the weight of an itemset in the suggested diagnosis. If *α=0*, all itemsets in that test image do not have even one match in the training representative association rules. Figure 5 shows the working principle of an AMIDE algorithm in condition 1. In this example, the values of (1) are: *nM*(*h*)=2 and *nN*(*h*)=1 for the itemset *h*={*Circum Benign*}. Therefore, if *w*_1_=(2/3)≥ *α*, the itemset *h*={*Circum Benign*} is returned by the algorithm.

#### Condition 2

**Figure 6:**
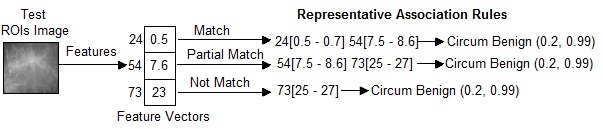
Example to show the calculation of Associative Medical Image Diagnosis Engine (AMIDE) in Condition 2.

An itemset *h* is returned by AMIDE in the suggested diagnosis, if the condition stated in (2) Figure 4 is satisfied, where *nP*(*h*) is the number of partial matches of the itemset *h*. The weight of the itemset *w*_2_ indicates the strength that an itemset belongs to the diagnosis. A threshold *δ(0<δ≤1)* is employed to limit the minimal number of matches required to return an itemset in the suggested diagnosis. If *δ=0,* all itemsets in that test image do not have even one match in the training representative association rules. Figure 6 shows the working principle of an AMIDE algorithm in condition 2. In this example, the values of (2) are: *nM*(*h*)=1, *nP*(*h*)=1, and *nN*(*h*)=1 for the itemset *h*={*Circum Benign*}. Therefore, if *w*_2_=(2/3)≥*δ*, the itemset *h*={*Circum Benign*} is returned by the algorithm, otherwise it is discarded.

If Condition 1 is satisfied with the threshold weight of *w*_1_, then the algorithm adds the diagnosis keyword *K* into *h*. If Condition 1 is not satisfied, then the algorithm AMIDE executes Condition 2 (see Figure 6). If it is satisfied with the threshold weight of *w*_2_, then the algorithm adds the diagnosis keyword *K* into *h*. Otherwise, the algorithm AMIDE does not add the diagnosis keyword *K* into *h*.

**Figure 7:**
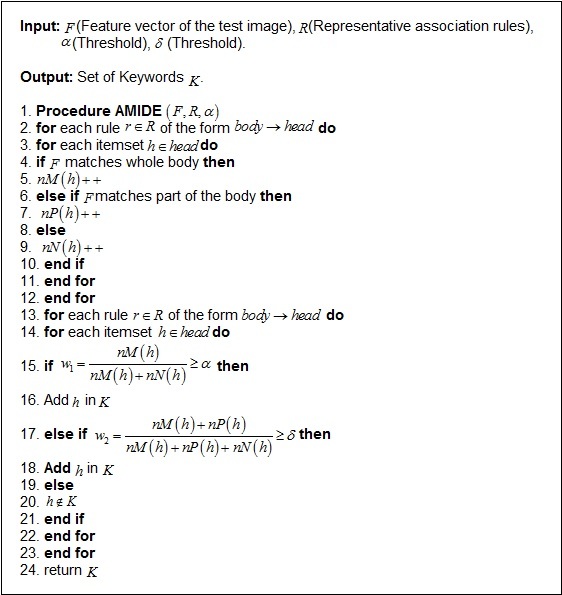
The AMIDE Algorithm.

Figure 7 summarizes the proposed AMIDE algorithm. As we show in the section on experiments, AMIDE is well-suited to generating suggestions for diagnoses. Although the approach presented here is applied to breast images, we describe the problem in a general way, in order to provide a common approach for other related fields. As we will show in the section on experiments, the proposed method is well-suited for medical images analysis, enhancing and bringing more confidence to the diagnosing process.

### Experimental Results

The dataset BI-RADS [60] consists of 446 abnormal images and 26 normal images taken from mammograms, collected from the Breast Imaging Reporting and Data System (BI-RADS) of the Department of Radiology, University of Vienna. Each image in BI-RADS has a diagnosis composed of three main parts.

- Morphology: Mass (circumscribed, indistinct, speculated); Arch. Dist.; Asym. Dens.; Calcifications (Amorph, Pleomorph, Linear, Benign).
- BI-RADS: Six levels (0-5).
- Histology: Benign lesions (breast tissue, cyst, calcifications, ductal hyperplasia, fibrosis, fibroadenoma, fatty tissue, hematoma, harmartomo, lymphangioma, lymphatic node, mastitis, mastopathia, papilloma, sclerosing adenosis and scar); high-risk lesions (atypical ductal hyperplasia, lobular carcinoma in situ, phyllodestumor and radial scar); and malignant lesions (ductal carcinoma in situ, invasive ductal cancer, invasive lobular cancer, invasive tubular cancer and muc. cancer).

The BI-RADS categorization was developed by the American College of Radiology to standardize mammogram reports and procedures. The BI-RADS categorization is summarized in Table 1.

The dataset mini-MIAS [61] used in our experiments is taken from the mini-Mammographic Image Analysis Society (mini-MIAS). It consists of 322 images and belongs to three big categories: normal, benign and malignant. There are 208 normal images, 63 benign, and 51 malignant, which are considered abnormal. In addition, the abnormal cases are further divided in six categories: micro calcification, circumscribed masses, speculated masses, ill-defined masses, architectural distortion, and asymmetry. All the images also include the locations of any abnormalities that may be present. The existing data in the collection consists of the location of the abnormality (like the center of a circle surrounding the tumor), its radius, breast position (right or left), type of breast tissues (fatty, fatty-glandular, and dense) and tumor type if it exists (benign or malign). All the mammograms show a medio-lateral oblique view.

For training purposes, we took 560 abnormal and 52 normal cases from both BI-RADS and mini-MIAS datasets. During the test phase, the developer and client invoked CIMIDx test cloud services model with the new images.

**Table 1:** BI-RADS^a^ assessment categorization.

Category	Description
0	Need additional imaging evaluation.
1	Negative.
2	Benign finding.
3	Probably benign finding. (Less than 2% malignant.) Short interval follow-up suggested.
4	Suspicious abnormality. (2-95% malignant.) Biopsy should be considered.
5	Highly suggestive of malignancy. (Greater than 2% malignant.) Appropriate action should be taken.

^a^Breast Imaging Reporting and Data System

## Results

Table 2 describes the classification accuracy of the proposed CIMIDx cloud services model for the diagnosis of the medical image. The diagnoses suggested by the CIMIDx method with the client test image were compared with the real diagnoses (biopsy results) of the training images (BI-RADS and mini-MIAS) given by specialists. To validate the CIMIDx prototype, we compared the CIMIDx (considering the diagnosis of calcifications (benign and malignant), masses (benign and malignant), and normal cases) with two well-known classifiers (Naïve Bayes and C4.5) and the IDEA [26] method. First, with Naive Bayes [62], the classifier is a probabilistic approach based on the Bayes’ theorem to predict the class labels. Second, with C4.5 [63], the classifier constructs a decision tree in the training phase, to derive the decision rules for testing a new mammogram image. Finally, with the IDEA method [26], a medical image diagnosis uses the representative association rules to predict the class label. The proposed CIMIDx framework leads to higher values of sensitivity, specificity, and accuracy, and it also presents the smallest error rates: false positive and false negative rates. During the training phase, the developer invoked the CIMIDx training cloud service model with BI-RADS [60] and mini-MIAS [61] datasets. Note that the training process was performed internally and produced representative association rules, that is, intelligent rules and deployed in the cloud for the diagnosis of new image.

**Figure 8:**
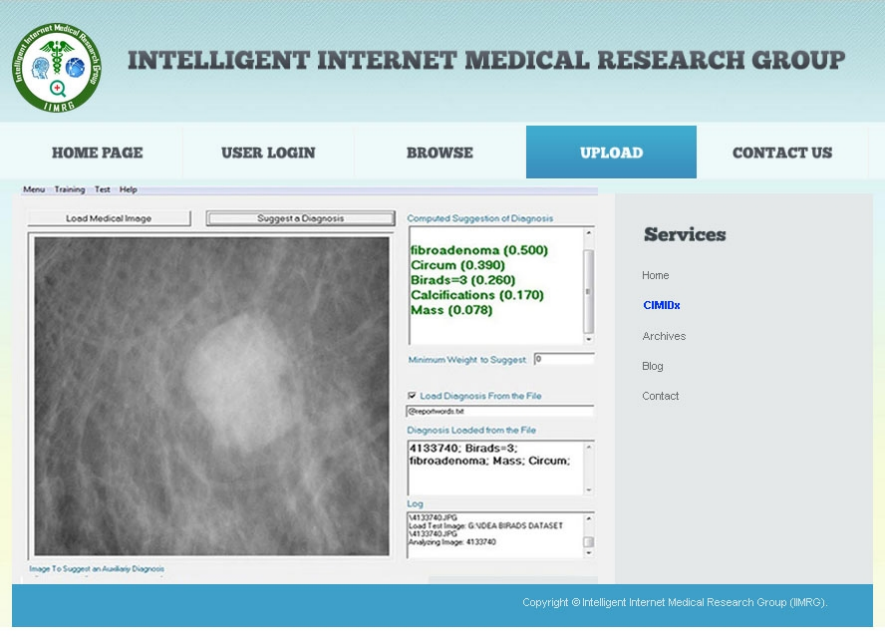
Implementation details of the CIMIDx framework.

The proposed CIMIDx method shows results of high sensitivity (up to 99.29%) and high accuracy (up to 98%). It is evident that the CIMIDx prototype is highly suitable for cloud-based self-management intervention for remote users. Figure 8 shows the implementation details of the cloud-based intelligent medial image diagnosis of the mammogram image.

Table 3 describes the characteristics of clients (patients and experts) in the use of CIMIDx for breast health. The CIMIDx is prompted by the detection of larger tumors, intermediate stage, low-grade DCIS, benign and normal cases. However, the proposed CIMIDx prototype-based breast cancer diagnosis can still be refined, using a greater number of samples (user images), and the results of this study show that its use can lead to the diagnosis of more cancers. This study was conducted by an experienced mammography interpreter (expert) during the developing stage of the CIMIDx prototype, and it is possible that the CIMIDx might contribute a higher percentage of increased cancer detection rates without biopsy information.

In our sample data collected from various hospitals and medical colleges, we found that the mean age of the CIMIDx users (patients) was 47.5 years (SD 33.2) and that of experts was 26 years (SD 18.3). The average length of time since the diagnosis was found to be more significant than the age. In the CIMIDx usage analysis for patients and experts for the diagnosis of breast cancer, the users were more educated, more likely to be younger, middle-aged group, and it differed neither in the breast cancer stages nor in the length of time since their cancer diagnosis. The annual household income and education levels are not reflected in the expert use of CIMIDx in the diagnosis of medical images. The stages of cancer diagnosis are broadly classified as normal, benign and malignant. The percentages of the subcategories of benign stages for CIMIDx users (patients) are given as normal breast issue 8% (8/97), fibrocystic disease 3% (3/97), fibroadenoma 6% (6/97), a typical ductal hyperplasia 3% (3/97), benign lesion, and others 1% (1/97). The percentages of the subcategories of malignant stages for CIMIDx users (patients) were given as DCIS grade I 9% (9/97), DCIS grade II and III 27% (26/97), IDC 25% (24/97), ILC 12% (12/97), ILC & IDC 3% (3/97), and malignant lesion, others 2% (2/97).

The cloud-based self-intervention system’s use is popular among breast cancer patients. Over 99% of our samples used it for breast health issues. The proposed CIMIDx results are consistent with those in prior literature, suggesting that a higher income and education are associated with patients’ information seeking [4,5]. We observe that the income and educational level were significant predictors for patients, of the use of CIMIDx for medical image diagnosis. However, the income and education level may not influence experts with the use of CIMIDx. Individuals with these characteristics may have been exposed to the new technologies, and may have the comfort level to experiment with the use of the CIMIDx prototype. In the CIMIDx study, the age, length of diagnosis time, and breast cancer stage were not significant predictors of cloud based self-intervention.

Table 4 shows the results of the logistic regression analysis for patients and experts in the diagnosis of medical images, using the CIMIDx prototype. From our test samples obtained from clients at various hospitals, health centers, and medical colleges, we found that the age and time since diagnosis were significant by .89. The annual household income (INR) for the category >2,70,000 was significant by .89, compared with the other income level categories. In education, grade 13-15 was significant by .96. In the benign stage, the category fibroadenoma was significant by .78, and in the malignant stage, the category ILC was significant by .86. The overall significance value *P* is obtained from the odds ratio (OR) and confidence interval (CI).

Table 5 shows the study between the two user groups based on the comparison of the intended versus observed frequency and activity: 44 low users and 53 high users. User feedback about the CIMIDx regarding ease of navigation was received as an input from the active users. The proportion of elements obtained as useful was higher in high users for the good category, 96% (51/53) compared to low users 88% (39/44) with a significance of *P*=.89.

The self-report by the users in the organization of information for the CIMIDx was gathered as input from the active CIMIDx users. The ratio of ingredients perceived was higher among the high users for the good category, 92% (49/53) compared to low users 93% (41/44) with a significance of *P*=.77.

Questionnaires about the CIMIDx’s usefulness were collected as active input from the users. The percentage of ingredients collected as useful was higher among the high users for the good category, 96% (51/53), compared to low users 95% (42/44), with a significance of *P*=.77.

The statistics about CIMIDx prototype’s user friendliness was received as active input from the users. The fraction of ingredients perceived as useful was higher among the high users 98% (52/53) compared to low users 98% (43/44) with a significance of *P*=.31.

Finally, the overall satisfaction of the prototype was gathered as input from the users. It was higher among the high users 98% (52/53) compared to low users 95% (42/44) with a significance of *P*=.31. The overall significance value *P* is based on the *t* tests and the Pearson chi-square tests.

**Table 2:** The classification accuracy of the proposed CIMIDx cloud services model with 150 client test images during the development of CIMIDx, and compared with the Naïve Bayes and C4.5 classification algorithms and IDEA method (n=150).^a^

Stages	Naïve Bayes		C4.5		IDEA Method		CIMIDx Method	
	Diagnosed	Missed	Diagnosed	Missed	Diagnosed	Missed	Diagnosed	Missed
	n (%)	n (%)	n (%)	n (%)	n (%)	n (%)	n (%)	n (%)
Normal breast issue	6 (4.0)	3 (2.0)	7 (4.7)	2 (1.3)	7 (4.7)	2 (1.3)	8 (5.3)	1 (0.7)
Fibrocystic disease	4 (2.7)	1 (0.7)	4 (2.7)	1 (0.7)	4 (2.7)	1 (0.7)	5 (3.3)	0
Fibro adenoma	7 (4.7)	3 (2.0)	8 (5.3)	2 (1.3)	9 (6.0)	1 (0.7)	9 (6.0)	1 (0.7)
Atypical ductal hyperplasia	4 (2.7)	1 (0.7)	4 (2.7)	1 (0.7)	5 (3.3)	0	5 (3.3)	0
Benign lesion, other	3 (2.0)	0	3 (2.0)	0	3 (2.0)	0	3 (2.0)	0
DCIS^b^, grade I	8 (5.3)	7 (4.7)	11 (7.3)	4 (2.7)	13 (8.7)	2 (1.3)	14 (9.3)	1 (0.7)
DCIS grade II & III	23 (15.3)	9 (6.0)	26 (17.3)	6 (4.0)	31 (20.7)	1 (0.7)	32 (21.3)	0
IDC^c^	42 (28.0)	3 (2.0)	42 (28.0)	3 (2.0)	44 (29.3)	0	44 (29.3)	0
ILC^d^	16 (10.7)	2 (1.3)	16 (10.7)	2 (1.3)	18 (12.0)	1 (0.7)	19 (12.7)	0
ILC & IDC	4 (2.7)	1 (0.7)	5 (3.3)	0	5 (3.3)	0	5 (3.3)	0
Malignant lesion, other	3 (2.0)	0	3 (2.0)	0	3 (2.0)	0	3 (2.0)	0
Total	120 (80.0)	30 (20.0)	129 (86.0)	21 (14.0)	142 (94.7)	8 (5.3)	147 (98.0)	3(2.0)

^a^At interviews with various medical colleges and hospitals in Chennai, Tamil Nadu, India, May 2013 to April 2014, the cloud-based system support intelligent medical image diagnosis prototype was used for breast health issues. The accuracy, sensitivity, specificity, false positive rate, and false negative rate results in percentage were calculated, with the true positive, true negative, false positive, and false negative measures.

^b^DCIS: ductal carcinoma in situ

^c^IDC: invasive ductal cancer

^d^ILC: invasive lobular cancer

**Table 3:** Characteristics of 150 women with breast cancer.

Demographic variable		Category	Use of CIMIDx by patients (n=97)	Use of CIMIDx by radiologist (n=53)	Significance (*P*)^a^
			mean (SD) or n (%)	mean (SD) or n (%)	
Age (years)			47.5 (33.2)	26 (18.4)	.53
Time since diagnosis (years)			47.5 (40.3)	26 (21.2)	.59
**Annual household income (INR)**					
		<1,00,000	12 (12.4%)		
		1,00,000-2,70,000	36 (37.1%)		
		>2,70,000	49 (50.5%)		
**Education**					
		Grades <12	13 (13.4%)		
		Grades 13-15	48 (49.5%)		
		Grades >15	36 (37.1%)		
**Stage**					
	**Normal**	Normal breast issue	8 (8.3%)	1 (1.9%)	.57
	**Benign**				
		Fibrocystic disease	3 (3.1%)	2 (3.8%)	
		Fibroadenoma	6 (6.2%)	4 (7.6%)	
		Atypical ductal hyperplasia	3 (3.1%)	2 (3.8%)	
		Benign lesion, other	1 (1.0%)	2 (3.8%)	
	**Malignant**				
		DCIS^b^, grade I	9 (9.3%)	6 (11.3%)	>.99
		DCIS grade II and III	26 (26.8%)	6 (11.3%)	
		IDC^c^	24 (24.7%)	20 (37.7%)	
		ILC^d^	12 (12.4%)	7 (13.2%)	
		ILC and IDC	3 (3.1%)	2 (3.8%)	
		Malignant lesion, others	2 (2.1%)	1 (1.9%)	

^a^At interviews with different medical colleges and hospitals in Chennai, Tamil Nadu, India, May 2013 to April 2014, the cloud-based system support intelligent medical image diagnosis prototype was used for breast health issues. The *P* values were calculated with *t* tests for the means, and the Pearson chi-Square tests for the percentages.

^b^DCIS: ductal carcinoma in situ

^c^IDC: invasive ductal cancer

^d^ILC: invasive lobular cancer

**Table 4:** Predictors of CIMIDx use of 150 women with breast cancer.

Stages		Category	Odds ratio	95% confidence interval	Significance (*P*)^a^
Age (years)			1.93	0.51-7.34	.89
Time since diagnosis (years)			0.19	0.07-0.53	.89
**Annual household income (INR)**					
		<1,00,000	1.00		
		1,00,000-2,70,000	2.44	0.19-31.53	.48
		>2,70,000	0.89	0.18-4.36	.89
**Education**					
		Grades <12	1.00		
		Grades 13-15	1.05	0.16-6.92	.96
		Grades >15	0.92	0.34-2.45	.86
**Stage**					
	**Normal**	Normal breast issue	1.00		
	**Benign**				
		Fibrocystic disease	0.5	0.01-19.56	.71
		Fibroadenoma	1.5	0.09-25.39	.78
		Atypical ductal hyperplasia	0.5	0.01-19.56	.71
		Benign lesion, other	-	-	.39
	**Malignant**				
		DCIS^b^, grade I	1.00		
		DCIS grade II and III	0.48	0.07-3.37	.45
		IDC^c^	1.33	0.32-5.59	.69
		ILC^d^	0.88	0.10-6.78	.86
		ILC and IDC	0.5	0.01-19.56	.71
		Malignant lesion, other	-	-	.39

^a^From interviews at various medical colleges and hospitals in Chennai, Tamil Nadu, India, May 2013 to April 2014, regarding the cloud-based system support intelligent medical image diagnosis prototype used for breast health issues, the *P* values were calculated with *t* tests for the means, and the Pearson chi-Square tests for the percentages.

^b^DCIS: ductal carcinoma in situ

^c^IDC: invasive ductal cancer

^d^ILC: invasive lobular cancer

**Table 5:** Group characteristics (social, economic, and the usefulness of the CIMIDx prototype for the two user groups).

Characteristics	Category	Users (n=97)		Significance (*P*)^c^
		Use of CIMIDx by low user (n=44)	Use of CIMIDx by high user (n=53)	
		mean (SD) or n (%)	mean (SD) or n (%)	
Age (years)		22 (22.6)	26.5 (31.8)	.89^a^
Time since diagnosis (years)		22 (24.0)	26.5 (33.2)	.89^a^
**Annual household income (INR)**				
	<1,00,000	4 (9.1)	6 (11.3)	.78^b^
	1,00,000-2,70,000	11 (25.0)	12 (22.6)	.48^b^
	>2,70,000	29 (66.0)	35 (66.0)	.89^b^
**Education**				
	Grades <12	5 (11.4)	8 (15.1)	.93^b^
	Grades 13-15	8 (18.2)	11 (20.7)	.96^b^
	Grades >15	31 (70.5)	34 (64.2)	.86^b^
**Ease of navigation**				
	Good	39 (88.6)	51 (96.2)	.89^b^
	Average	5 (11.4)	2 (3.7)	.81^b^
**Organization of information**				
	Good	41 (93.2)	49 (92.5)	.77^b^
	Average	3 (6.8)	4 (7.5)	.81^b^
**Usefulness**				
	Good	42 (95.5)	51 (96.2)	.77^b^
	Average	1 (2.3)	2 (3.7)	.39^b^
**User friendliness**				
	Good	43 (97.7)	52 (98.1)	.31^b^
	Average	1 (2.3)	1 (1.9)	.99^b^
**Overall satisfaction**				
	Good	42 (95.5)	52 (98.1)	.31^b^
	Average	2 (4.5)	1 (1.9)	.39^b^

^a^ *t* test

^b^Pearson chi-square test

^c^From the interviews at various medical colleges and hospitals in Chennai, Tamil Nadu, India, May 2013 to April 2014, regarding the cloud-based system support intelligent medical image diagnosis prototype used for breast health issues, the *P* values were calculated with *t* tests for the means, and the Pearson chi-Square tests for the percentages.

## Discussion

### Principal Findings

In this paper, we introduced the CIMIDx prototype that follows a new approach to a systematically developed and well-organized diagnosis of mammogram images for authorized patients and experts. We performed several experiments to validate the proposed CIMIDx method and the results show the quantitative evaluation that users positively assessed and searched the functionalities in an efficient manner.

The objective of the CIMIDx technology is to provide the facility for experts and low-income patients, anywhere in the world at any time to use the CIMIDx prototype-based mammogram image diagnosis. The results show a high sensitivity of up to 99.3% (139/140) and accuracy of up to 98% (147/150) (the classification accuracy up to 99.1% (117/118), in the malignant mass; up to 96% (22/23), in the benign; and up to 89% (8/9), in the normal category).

The CIMIDx prototype increased the cancer diagnosis rate for the benign (mass and calcification) stages by 96% (22/23), compared with the other methods (from 91% (21/23), of IDEA method; 83% (19/23), by C4.5; and 78% (18/23), by Naïve Bayes). Similarly, it increased the diagnosis rate of malignant (mass and calcification) stages by 99.2% (117/118) (from 96.6%, 114/118, of the IDEA method; 87.3% (103/118) by the C4.5; and 81.4% (96/118), by the Naïve Bayes).

The CIMIDx alone diagnosed 98.0% (147/150) of benign and malignant stages, whereas the IDEA method diagnosed 94.7% (142/150), the C4.5 86.0% (129/150), and the Naïve Bayes 79.3% (119/150). In total, 2.0% (3/150) were dismissed by the CIMIDx whereas the IDEA method dismissed 5.3% (8/150), the C4.5 14.0% (21/150), and the Naïve Bayes 20.7% (31/150), which is clear from Table 2.

The characteristics of patients and experts have been discussed in the use of the CIMIDx prototype for breast health. In our samples, 64.7% (97/150) of patients and 35.3% (53/150) of experts used the CIMIDx for cloud-based diagnosis of the breast cancer image. Of the 150 women screened during the study period, 23 were diagnosed under the category of benign stage, 118 were diagnosed under malignant category, and 9 were diagnosed under normal category. Out of the 150 test images 15.3% (23/150) (benign stages, the patients diagnosed by the CIMIDx prototype were 100%, 13/13) and those by experts were 90% (9/10). Out of 150 test samples, 78.7% (118/150) malignant stages, the patients diagnosed by the CIMIDx prototype were 99% (75/76), and those by experts diagnosed were 100% (42/42). Out of 9/150 (6.0%) normal stage cases, the patients diagnosed by the CIMIDx prototype were 88% (7/8), and those by experts were 100% (1/1). It is evident from Table 3 that the malignant stage of diagnosis was significant by >.99 than the benign stage.

The predictors of the CIMIDx used 150 women with breast cancer, and obtained the results based on the logistic regression analysis. As can be seen, the income and education levels remained significantly related in the diagnosis of the medical image with the CIMIDx prototype. Those with an income level (INR) >2,70,000 have higher significance than people with incomes between 1,00,000-2,70,000 and <1,00,000. Patients with post graduate education (ie, grades >15) have higher significance than those with undergraduate education (ie, grades 13-15) and high school level (ie, grades <12). The use of the CIMIDx prototype is unrelated to the patients’ age, duration of the diagnosis, and breast cancer stages. It is evident from Table 4 that the model was significant with χ^2^_0.90_=0.0164 and *P*=.89.

In this study, the user (low and high) groups differed only in the usage statistics, which is how they were classified. With regard to how the intervention was used, high users provided the self-help information more often and reported more consistently on the social and economic and the usefulness of ingredients compared to low users. In addition, no specific sociodemographic, medical, or personal characteristics were found that distinguished the user groups, supporting our hypothesis that the present generic, fully automated intervention could be acceptable for patients’ use of the CIMIDx prototype. It is evident from Table 5 that the CIMIDx prototype was highly useful with the significance of *P*=.77.

### Strengths and Limitations

The strengths of our study include the high participation rate and the inclusion of those with different stages of cancer. However, we relied on self-reports, and this information may require further refining of the CIMIDx prototype in a better manner. The use of the CIMIDx prototype provided the results of the test image with biopsy relevant information. In [49], the author discussed that 70% of the physicians refer their cancer patients to various online support services for their cancer diagnosis. Breast cancer patients’ use of the CAD services is quite low in the earlier stages, from 2% to 8%. Of those patients aware of cloud-based cancer information services, which they found to be 7%, only one-half (7%) used it. The diagnosis is limited to those with early-stage breast cancer, women <60 years, and those with a diagnosis of almost 6 months’ period. It is possible that many of these late-stage patients died during the time interval from diagnosis to the study completion or refused to participate. For those recently diagnosed, improved mammography screening rates allow many to be diagnosed with an early-stage rather than a late-stage cancer. Furthermore, the participants were from anywhere in the world. Many patients may find it more comfortable to seek information over the cloud-based medical image diagnosis (self-evaluation), than to use traditional cancer support services. The research should evaluate whether patients and/or experts feel that there are potential clinical benefits for this CIMIDx use. The refinement of the CIMIDx is based on the patient information from the questionnaire in Multimedia Appendix 1.

### Conclusions

This study shows that women felt favorably about the use of the cloud-based self- management website for breast cancer survivors to meet their expectations for credibility, accuracy, privacy, and sensitivity to their situation. The present study mainly focuses on the implementation and usage evaluations of the generic, fully automated cloud-based self-intervention for breast health issues. The proposed CIMIDx prototype is an efficient and useful tool for the medical and scientific communities, in order to manage mammographic images including their associated diagnosis, featuring the advantages and functionalities of a cloud service. This study demonstrated that applying the CIMIDx prototype to experts, resulted in the detection of more cancers in screening and diagnosing patients, with an increased sensitivity of up to 99.3% and accuracy rate of up to 98%. This study focuses on the evaluations of the usage statistics for the CIMIDx prototype in the realistic estimation of exposure to the intervention of clients.

## Multimedia Appendix 1

CIMIDx questionnaire.

## Abbreviations

AMIDE: Associative Medical Image Diagnosis Engine
AOI: area of interest
BI-RADS: Breast Imaging-Reporting and Data System
CADe: computer-aided detection
CADx: computer-aided diagnosis
CC: central controller
CIMIDx: Cloud-Based System Support Intelligent Medical Image Diagnosis
DCIS: ductal carcinoma in situ
EC2: Amazon Elastic Compute Cloud
FAB: forward-and-backward
HTTP: Hypertext Transfer Protocol
HTTPS: Hypertext Transfer Protocol Secure
ICT: information communication technology
IDC: invasive ductal cancer
ILC: invasive lobular cancer
LSF: level set function
LSM: level set method
MICAS: Medical Image Collaborative Analysis System
mini-MIAS: mini-Mammographic Image Analysis Society
ROI: region of interest
SC: service consumer
SD: service discovery
SNR: signal-to-noise ratio
SOAP: Simple Object Access Protocol
SP: service provider
SRG: seeded region growing
UDDI: Universal Description, Discovery and Integration
WSDL: Web Services Description Language
XML: Extensible Markup Language
