# Supplementary material for: CIMIDx: Prototype for a Cloud-Based System to Support Intelligent Medical Image Diagnosis With Efficiency
Source: JMIR Med Inform. 2015 Mar 27;3(1):e12. doi: 10.2196/medinform.3709 (PMC4393505; doi:10.2196/medinform.3709)
Supplement: Supplementary file 1 [file medinform_v3i1e12_app1.pdf]

Multimedia Appendix I

INTELLIGENT INTERNET MEDICAL RESEARCH GROUP (IIMRG)

INFORMATION AND COMMUNICATION ENGINEERING RESEARCH

ANNA UNIVERSITY, CHENNAI, TAMILNADU, INDIA.

Dear Participant:

The purpose of this questionnaire is to evaluate Cloud-Based System Support Intelligent Medical Image Diagnosis (CIMIDx) framework from your perspective. Kindly answer all the questions. All information provided in this questionnaire is confidential and only for research purposes. If you have any enquiries regarding this questionnaire, you can contact us at senthil@cs.annauniv.edu and rv_bhavani@yahoo.com.

Q.NO	QUESTION	ANSWER
1.	Category of your age group. (Circle one)<br>A. 30-40 B. 40-50 C. 50-60 D. 60-70<br>If no, you now furnish your age_____.	**A B C D**
2.	Do you use the Internet (circle one)<br>If no, you have now finished<br>If yes, continue below:<br>Internet access (circle as many as relevant)<br>A. Home B. Work C. Internet Café D. Friend	**YES NO**<br><br><br><br>**A B C D**
3.	How frequently do you use the Internet for your personal care?<br>A. Weekly B. Monthly C. Half yearly D. Yearly	**A B C D**

4. Do you use the World Wide Web (circle one) **YES NO**

If yes, do you use it for information regarding breast health / women's health issues? **YES NO**

If yes, estimated **current** number of hours weekly ____.

If yes, estimated number of hours weekly **before surgery_____.**

5. Do you use e-mail (circle one) **YES NO**

If yes, do you use it for information regarding breast health / women's health issues? **YES NO**

If yes, estimated **current** number of messages weekly__.

If yes, estimated number of messages weekly **before surgery___.**

6. Categorize your annual income (Circle one) **A B C**

A. < 1,00,000 B. 1,00,000 – 2,70,000 C. 2,70,000

7. Does the CIMIDx prototype provide ease of navigation? **YES NO**

8. Do you think that the information available with CIMIDx for breast health / women's health issues is organized? **YES NO**

9. Do you feel that the cloud based medical diagnosis is really useful for society? **YES NO**

10. Is the cloud based medical diagnosis framework user friendly? **YES NO**

11. Does the CIMIDx user get overall satisfaction? **YES NO**

12. Are you interested in self-diagnosis about breast health / women's health issues if the cloud based medical diagnosis system facility provided in the Internet before consulting a physician? **YES NO**

13.	Are you interested to discuss your breast health / women’s health issues in the Internet blogs, after testing from the cloud based medical diagnosis system?	**YES**	**NO**
14.	Do you think that Internet blogs creates a lot of awareness for the breast health / women’s health issues	**YES**	**NO**
15.	Do you feel that your using the cloud Internet based medical diagnosis for breast health / women’s health issues can considerably save time and money?	**YES**	**NO**

Name of the Participant:

Signature of the Participant:
